# Direct Electron Transfer of Dehydrogenases for Development of 3rd Generation Biosensors and Enzymatic Fuel Cells

**DOI:** 10.3390/s18051319

**Published:** 2018-04-24

**Authors:** Paolo Bollella, Lo Gorton, Riccarda Antiochia

**Affiliations:** 1 Department of Chemistry and Drug Technologies, Sapienza University of Rome P.le Aldo Moro 5, 00185 Rome, Italy; paolo.bollella@uniroma1.it; 2 Department of Biochemistry and Structural Biology, Lund University, P.O. Box 124, 221 00 Lund, Sweden; lo.gorton@biochemistry.lu.se

**Keywords:** dehydrogenases, direct electron transfer, cellobiose dehydrogenase, fructose dehydrogenase, biosensors, biofuel cells

## Abstract

Dehydrogenase based bioelectrocatalysis has been increasingly exploited in recent years in order to develop new bioelectrochemical devices, such as biosensors and biofuel cells, with improved performances. In some cases, dehydrogeases are able to directly exchange electrons with an appropriately designed electrode surface, without the need for an added redox mediator, allowing bioelectrocatalysis based on a direct electron transfer process. In this review we briefly describe the electron transfer mechanism of dehydrogenase enzymes and some of the characteristics required for bioelectrocatalysis reactions via a direct electron transfer mechanism. Special attention is given to cellobiose dehydrogenase and fructose dehydrogenase, which showed efficient direct electron transfer reactions. An overview of the most recent biosensors and biofuel cells based on the two dehydrogenases will be presented. The various strategies to prepare modified electrodes in order to improve the electron transfer properties of the device will be carefully investigated and all analytical parameters will be presented, discussed and compared.

## 1. Introduction

Dehydrogenase enzymes (E.C. 1.1.1.-1.1.2) belong to the group of oxidoreductases, which oxidise a substrate by reducing an electron acceptor. Hundreds of dehydrogenases occur in nature and most of them are NAD(P) (nicotinamide adenine dinucleotide (phosphate), acting as electron acceptor/donor) dependent [1]. In this case the cofactor is not covalently bound to the enzyme but it must be present in the reaction medium to activate the biocatalytic function of the enzyme itself, by acting as a carrier of two electrons and one proton. By contrast, all other dehydrogenases require for their activity a cofactor tightly bound to the protein, which can be either FAD (flavin adenine dinucleotide), FMN (flavine mononucleotide), PQQ (pyrroloquinoline quinone) [2] or Moco (molybdopterin) [3,4]. These enzymes are also called pyridine nucleotide-independent dehydrogenases, as they do not use NAD^+^ or NADP^+^ (nicotinamide adenine dinucleotide phosphate) as electron acceptor [1].

Dehydrogenases can therefore be divided into four main classes, depending on the type of cofactor: (i) NAD^+^/NADP^+^ dependent; (ii) flavin (FAD or FMN) dependent; (iii) PQQ dependent; and (iv) Moco dependent enzymes.

The thermodynamic redox potential of NAD^+^/NADH is −0.52 V versus Ag|AgCl_sat_ at pH 7 [5,6], determined by using potentiometric titration [7]. The NADH cofactor itself is not a useful redox electron shuttle in bioelectrochemistry, because of a high electrochemical overpotential, required to drive the electron transfer reaction and lack of chemical reversibility for the NADH/NAD^+^ redox process and because of the interfering adsorption of the cofactor itself and reaction products at electrode surfaces [5]. For these reasons, NAD^+^ dependent dehydrogenase based biosensors were realised by the introduction of various redox mediators [8,9,10] or of diaphorase (NADH:acceptor oxidoreductase), a redox enzyme, which catalyses the oxidation of NADH with the concomitant reduction of a redox mediator. Several 2nd generation biosensors were described by using the two-enzyme cascade NAD(P)-dependent dehydrogenase/diaphorase [11,12,13,14].

The FAD, FMN PQQ, Moco cofactors show thermodynamic redox potentials in the potential window of about −0.26 and +0.14 V vs Ag|AgCl_sat_ at pH 7, respectively [9,15], which are less negative than that for the NAD^+^/NADH couple. In general biosensors based on FAD-, FMN-, PQQ- and Moco-dependent dehydrogenases require the use of redox mediators to shuttle the electrons between the active site and the electrode [16,17,18,19]. Many examples of 2nd generations biosensors by employing different mediators were reported in the literature based on FAD dependent [20,21,22] and PQQ-dependent dehydrogenases [23,24,25,26,27,28].

Several dehydrogenases harbouring FAD, FMN, PQQ and Moco as cofactor can contain an additional heme prosthetic group [29]. Therefore they can in turn be divided into two categories: flavoproteins/flavohemoproteins, quinoproteins/quinohemoproteins, molybdopterin/ molybdopterinhemoproteins respectively, depending on whether they contain the heme group or not. In the case of also containing a heme the FAD, FMN, PQQ or Moco containing domain may be connected via the heme containing cytochrome domain to an electrode and thus the cytochrome domain acts as “a built in mediator” [30].

Such (FAD, FMN, PQQ, or Moco dependent) dehydrogenases have been investigated as potential enzymes that can undergo direct electron transfer (DET) with electrodes [29]. Recent studies indicate that efficient DET is hard to obtain in the absence of the additional heme group (quinohemoprotein and flavohemoprotein). The catalytic FAD, FMN, PQQ, or Moco containing dehydrogenase domain is structurally connected in various ways to the heme containing cytochrome domain thereby allowing smooth electron transfer from the catalytic site via the heme to the external electron acceptor (electrode). However, the connection between the two domains may be very sensitive to a series of parameters such as pH, ionic strength, concentration of specific cations, determining the distance between the two domains and thus the rate of the internal electron transfer.

Due to their superior electron transferability, such heme-containing, multicofactor dehydrogenases, consisting of a FAD-, FMN-, PQQ-, or Moco-harbouring catalytic subunit/domain and a heme-containing electron transfer subunit/domain, were recently shown to undergo DET with several different electrode surfaces [31,32,33,34,35,36,37,38,39,40,41,42] as initially was pointed out by Ikeda et al. already in the early 1990s [30,43].

In this review we focus our attention on two dehydrogenases that have been extensively discussed with data reported in the literature for their ability to undergo efficient DET: cellobiose dehydrogenase (CDH) and fructose dehydrogenase (FDH). CDH and FDH are gaining increased attention in the last few years as catalysts for the oxidation of lactose/glucose and fructose in several biosensing and biofuel cells (BFCs) applications [44,45,46,47,48,49,50,51,52,53].

In the following sections, the DET mechanism of the two dehydrogenases is described and an analytical comparison of the electrochemical performances of various CDH and FDH based biosensors and BFCs are reported. For all electrochemical systems investigated, information is given on the type of electrode modification performed, electrode assembly, electrode platform and device stability. Analytical parameters such as LOD, sensitivity, dynamic linear range for biosensing applications and OCV and power for BFC applications are also reported.

## 2. Direct Electron Transfer of Dehydrogenases

**Figure 1:**
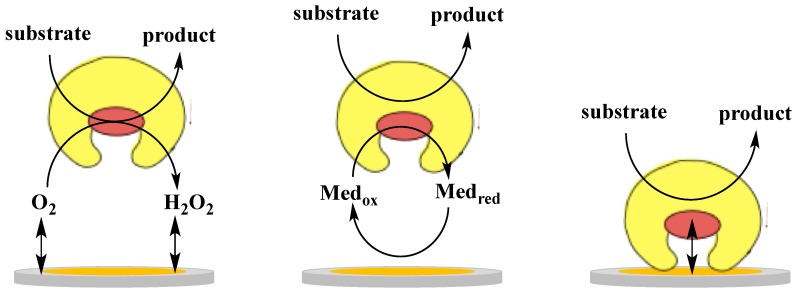
Schematic representation of electron transfer in biosensors: 1st generation (**left**), 2nd generation with soluble or immobilised mediator (**centre**) and 3rd generation (**right**).

The electronic coupling between redox enzymes and electrodes for the development of analytical devices such as biosensors and BFCs can be realised according to three mechanisms, which in turn are denoted 1st, 2nd and 3rd generation biosensors [29]: (i) electroactivity of the substrate or product of the enzymatic reaction (1st generation); (ii) mediated electron transfer (MET) with the use of redox mediators, small electroactive molecules (freely diffusible or bound to side chains of flexible redox polymers), which are able to shuttle the electrons between the enzyme active site and the electrode (2nd generation); (iii) DET between the redox centre of the enzyme and the electrode surface (3rd generation). Figure 1 shows a schematic representation of the three mechanisms of electronic communications between enzyme and electrode. Commercial biosensors are mostly based on either 1st or 2nd generation biosensors [29], however, recent progress in bioelectrochemistry in combination with bioengineering of enzymes have shown that 3rd generation biosensors have now reached the market [54].

In particular, when discussing the 2nd generation sensors we should distinguish between a freely soluble monomeric mediator (e.g., Fe(CN)_6_^3−/4−^, quinones) and an immobilised mediator in the form of for example, a redox polymer equipped with Os^3+/2+^-complexes, quinones, or other redox compounds that can be co-immobilised with the enzyme making reagentless biosensors possible. For biosensor development, the 3rd generation format based on DET mechanism shows important advantages compared to MET, 2nd generation biosensors with a soluble or polymer bound mediator: first of all the absence of a mediator allows a higher selectivity, because the biosensor can operate at a potential closer to the formal potential (E°’) of the redox enzyme, thus reducing possible interfering reactions. Moreover, both soluble and polymer bound mediators may also mediate unspecific reactions and the lack of a reagent in the reaction sequence makes the device easier to realise. However, as mentioned adsorbed/immobilised mediators allow the making of reagentless biosensors possible (no freely diffusing mediator in solution), which is an obvious advantage compared to other 2nd generation biosensors that rely on the addition of a mediator to the sensing solution [55,56]. This type of bound mediators can drive catalytic processes also at low overpotentials by modifying the molecular structure, thus tuning the E°’ of the redox polymer [57]. Finally, 3rd generation biosensors based on DET offer a wide range of possibility to modulate the desired properties of the analytical device using protein modification either through gene or protein engineering techniques [58] or new interfacial technologies making use of nanomaterials (e.g., metal nanoparticles and carbon based nanomaterials) [59].

Also for development of BFCs, DET possesses some important advantages over MET [60]. The use of redox mediators usually leads to voltage losses arising from the potential difference between the active site of the enzyme and the mediator. Mediators for anodic reactions show redox potentials at least 90–200 mV higher than those of the oxidative redox enzymes, thus decreasing the potential difference between the BFC anode and the cathode. Nevertheless, highly efficient BFCs obtained also with lower potential differences are reported in the literature [61]. Moreover, a DET design allows several simplifications in the construction of sugar/O_2_ BFCs based on oxygen insensitive dehydrogenases, as no membranes and no separate compartments are necessary, thus facilitating the miniaturisation process. One of the main drawbacks of using DET communication in constructing BFCs is the difficulty to electrically connect a sufficient amount of enzyme molecules, which can limit the efficiency and the power output of the devices [60].

Although DET presents favourable characteristics for both biosensors and BFCs, only a few groups of enzymes are found to be capable of interacting directly with an electrode via a DET mechanism. This is because the redox centres of the enzymes are often buried within the protein structure in combination with a lack of an electron transfer pathway connecting the active site with the protein surface [60,62,63,64,65,66,67,68,69,70,71]. An efficient DET mechanism has been demonstrated for a restricted number of enzymes [60,63,64,66,67], such as multicopper blue oxidases (laccases and bilirubin oxidases) [72,73,74], hydrogenases [64,70,71,74,75], peroxidases [29,76,77] and a few dehydrogenases, all containing metallocentres and/or one or more heme groups [63,65,66,67,78]. As for the dehydrogenases, DET communication has been reported also for a limited number of heme containing enzymes, similar in their multicofactor composition (FAD, FMN, PQQ, or Moco in combination with heme) to dehydrogenase enzymes. However, these enzymes exhibit a medium-high reactions rate with molecular oxygen and are therefore denoted oxidases rather than dehydrogenases. These are reported in Table 1. All of them are FAD, FMN, PQQ, or Moco containing heme proteins [32,45,47,48,79]. Some even containing Fe-S clusters such as membrane bound succinate dehydrogenase (complex II in the respiratory chain), which shows DET properties both in its isolated “native” state [80] as well as in its truncated form for which the heme containing part was removed [81]. Among them, CDH and FDH are the most widely investigated for biosensing and BFCs applications and for this reason they will be treated separately below.

## 3. Cellobiose Dehydrogenase

Cellobiose dehydrogenase (CDH; E.C 1.1.99.18) is an extracellular enzyme, belonging to the oxidoreductase group, secreted by wood-degrading, phytopatogenic and saprotrophic fungi within the phyla of Basidiomycota and Ascomycota [93,94]. In 1974, Westermark and Eriksson reported for the first time about the enzymatic activity in *Trametes versicolor* and *Phanaerochaete chrysosporium* observing the oxidation of guiacol while adding cellobiose [95]. During the last 40 years, a lot of efforts have been addressed to elucidate the full electron transfer mechanism, enzyme catalytic activity towards several substrates (cellobiose, lactose, glucose, etc.), enzyme structure and bioengineering pathways to adapt the enzyme to some commercial/industrial purposes [44,83].

CDH belongs to the flavohemoprotein family, which includes also mandelate dehydrogenase, fumarate dehydrogenase, bacterial cytochrome P-450, nitric oxidase synthase and flavocytochrome *b*_2_ [93]. Unlike these enzymes, CDH is the only extracellular one. It contains two subunits/domains: subunit I is the catalytic dehydrogenase domain (DH_CDH_) similar to the DH domain of other oxidoreductases belonging to the glucose-methanol-choline (GMC) oxidoreductase superfamily with a flavin adenine dinucleotide (FAD) co-factor non-covalently bound to the enzyme structure; subunit II is the cytochrome domain (CYT_CDH_), which contains a heme *b* and in bioelectrochemistry acts as “a built-in mediator” by facilitating the shuttling of the electrons to an electrode [96,97]. Both subunits are connected through a flexible linker responsible for the modulation of the rate of the internal electron transfer (IET) reaction by varying the experimental conditions such as changing the pH, ionic strength or the concentration of divalent cations [98,99,100]. The oxidation of the natural substrate cellobiose fully reduces FAD in a 2e^−^/2H^+^ process and the electrons are sequentially transferred one by one through the IET pathway to the CYT_CDH_ resulting in reduced heme *b*, which in turn finally donates the electrons to the electrode [101,102].

Based on the phyla of fungi secreting CDH, it is possible to divide them into two classes: class I and class II, from Basidiomycota and Ascomycota, respectively. The CDHs belonging to class I exhibit a shorter enzyme sequence and a lower molecular weight (c.a. 80 kDa) compared to those of class II as well as a highly conserved linker region, an acidic working pH (3.5–4.5) and a poor oxidizing capacity towards glucose and other monosaccharides. Conversely, class II CDHs are able to oxidise both disaccharides and monosaccharides, exhibit a more complex and longer enzyme sequence (including also a cellulose binding module), a higher molecular weight (c.a. 115 kDa) and some have a neutral working pH (7.4) [45,103].

**Figure 2:**
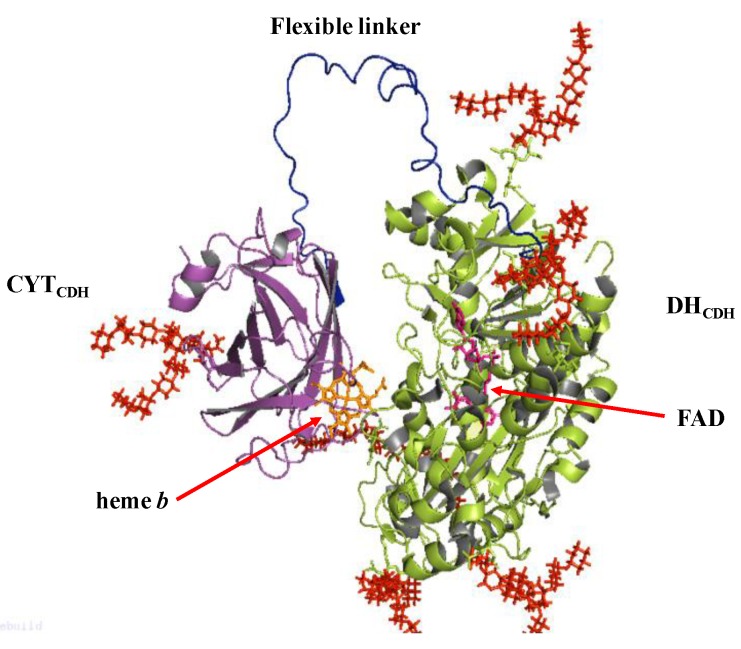
Schematic representation of cellobiose dehydrogenase (CDH): DH_CDH_ domain is shown in green with the FAD cofactor in pink; CYT_CDH_ domain in violet with heme *b* cofactor in orange; the flexible linker, in blue, is responsible for the modulation of internal electron transfer (IET); all the potential glycosylation sites are shown in red.

The crystal structure of the separated domains of CDH has been reported for the first time by the group of Divne et al. for *Phanerochaete chrysosporium* (*Pc*CDH) considering the CYT_CDH_ domain (PDB 1D7C) and DH_CDH_ domain (1KDG) separately [104,105], as shown in Figure 2. The crystal structure of CYT_CDH_ showed a sandwich fold of two antiparallel β sheets with an exposed heme *b* cofactor, like in other cytochromes. The heme *b* is hexacoordinated so that the propionate residues are sufficiently exposed and affecting the internal electron transfer (IET). Only one crystal structure is available for the whole enzyme, extracted from *Neurospora crassa* and published by the same group recently [106]. The natural electron acceptor to CDH, lytic polysaccharide monooxygenase (LPMO) was only recently discovered [107] revealing the central role of CDH in the degradation of lignocellulose in nature [108,109].

**Figure 3:**
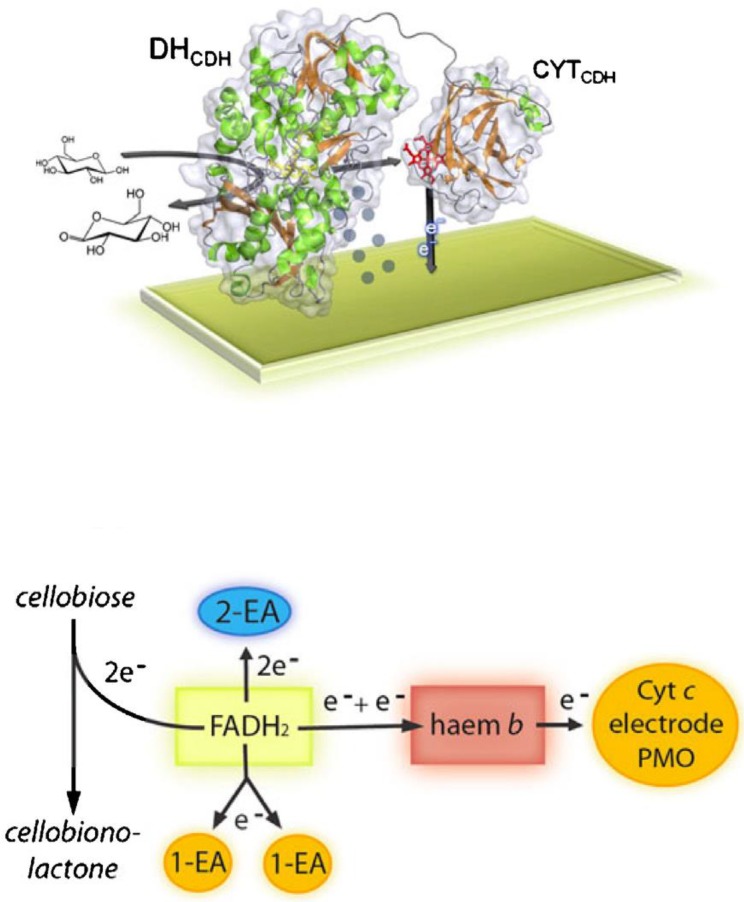
Electron transfer pathways from the substrate through CDH to various electron acceptors. One-(1-EA) and two-electron acceptors (2-EA) can be reduced directly by FADH_2_ in the DH_CDH_. Alternatively, electrons can be transferred by IET to heme *b* in the CYT_CDH_, which works as a relay for the reduction of macromolecular electron acceptors like polysaccharide monooxygenase (PMO), cyt *c* or an electrode. Figure 3 is reproduced from [89] published as open-access paper in Analytical and Bioanalytical Chemistry edited by Springer-Verlag.

The electron transfer reaction between the DH_CDH_ domain and an electrode can occur mainly according to two different routes depending on the absence (DET based reaction) or the presence of a redox mediator (MET based reaction) with a suitable redox potential [82], as schematised in Figure 3.

In the presence of any electron acceptor, an aldose is oxidised at the C1 position (only the β-D-anomer is a substrate for CDH) into its corresponding lactone and concurrently FAD in the active site of the DH_CDH_ is fully reduced to FADH_2_-DH_CDH_:

(1) $$aldose+FAD-DH_{CDH}\to lactone+FADH_{2}-DH_{CDH}$$

The reoxidation of FADH_2_-DH_CDH_ can follow two different reaction mechanisms:

(1) The electrons are transferred from FADH_2_ to the electrode directly if a 2e^−^, 2H^+^ acceptor is employed, such as a quinone (Q) or an equivalent aromatic redox compound [82]:

(2) $$FADH_{2}-DH_{CDH}+Q\to FAD-DH_{CDH}+QH_{2}$$

The reduced quinone, QH_2_, is then reoxidized at the electrode at an applied potential (E_app_) higher than the E°’ of the Q/QH_2_ redox couple, $E_{Q/QH_{2}}^{0’}$

(3) $$QH_{2}\overset{E_{app}>E_{Q/QH_{2}}^{0’}}{\to }Q+\;2H^{+}+2e^{-}$$

(2) the electrons are transferred from FADH_2_ to the electrode directly and sequentially if a 1e^−^, non-H^+^ acceptor, is employed, such as an Os^3+^-complex (Os^3+^) [83]:

(4) $$FADH_{2}-DH_{CDH}+Os^{3+}\to FADH\cdot -DH_{CDH}+Os^{2+}+H^{+}$$

(5) $$FADH\cdot DH_{CDH}+Os^{3+}\to \;FAD-DH_{CDH}+Os^{2+}+H^{+}$$

The Os^2+^ ions formed are reoxidized at the electrode if E_app_ is higher than the E°’ of the Os^3+/2+^ redox couple, $E_{Os^{3+/2+}}^{0’}$:

(6) $$2Os^{2+}\overset{E_{app}>E_{Os^{3+/2+}}^{0’}}{\to }2Os^{3+}+2e^{-}$$

In the absence of a mediator the electrons can be transferred from FADH_2_-DH_CDH_ to the CYT_CDH_ sequentially in an internal electron transfer process (IET)

(7) $$FADH_{2}-DH_{CDH}-CYT_{CDH}\overset{IET}{\to }FADH\cdot -DH_{CDH}-CYT_{CDH}\cdot^{-}+H^{+}$$

The reoxidation of the reduced enzyme can be summarised as follows:

(8) $$FADH-DH_{CDH}-CYT_{CDH}\overset{1{}^{\circ}\;IET\;step}{\to }FAD\cdot -DH_{CDH}-CYT_{CDH}\cdot^{-}$$

This step is followed by a first electron transfer (DET) step to the electrode, which is immediately followed by a second IET step delivering the second electron from the DH_CDH_ to the CYT_CDH_:

(9) $$FAD\cdot -DH_{CDH}-CYT_{CDH}\cdot^{-}\overset{1^{st}ET\;to\;the\;electrode\;and\;2^{nd}\;IET\;step}{\to }FAD-DH_{CDH}-CYT_{CDH}\cdot^{-}+electrode$$

Finally, the second electron is delivered to the electrode:

(10) $$FAD-DH_{CDH}-CYT_{CDH}\cdot^{-}\overset{\;2^{nd}\;ET\;to\;the\;electrode}{\to }FAD-DH_{CDH}-CYT_{CDH}+e^{-}+electrode$$

Various CDHs have been extensively utilised over the past twenty years to develop lactose/glucose biosensors and enzymatic fuel cells [44,82,83], which are carefully reviewed in the following two sections, highlighting the results achieved in terms of sensitivity, selectivity, linear range, stability, operational voltage and maximal power output.

### 3.1. Cellobiose Dehydrogenase Based Biosensors

As mentioned above, CDH can be classified into two groups: class I CDH, only catalysing cellodextrins and lactose efficiently and class II, which can catalyse not only cellodextrins and lactose but may also the oxidation of monosaccharides like glucose and other low molecular weight dextrins such as maltose and so forth, although the turnover number is higher for cellodextrins and lactose than for example, for glucose. Therefore in this review the most recent applications of CDH based biosensors for lactose and glucose will be reported separately (Table 2).

#### 3.1.1. CDH Based Lactose Biosensors

Before the discovery of the DET properties of CDH biosensors for lactose were typically based on combining lactase, hydrolysing lactose into glucose and galactose, with either glucose oxidase (GOx) or galactose oxidase [116,117,118,119]. Novel electrochemical biosensors for lactose detection based on class I CDHs were initially based on simple adsorption onto plain graphite electrodes [96] or covalent immobilisation on premade screen printed carbon based electrodes [110,120] used with success for lactose detection in the dairy industry [119,121]. Also screen printed electrodes (SPEs) have been considered by Safina et al. to develop portable lactose biosensors based on two different class I CDHs, *T. villosa* CDH and *P. sordida* CDH [110]. They used two different SPEs: modified with multiwalled carbon nanotubes (MWCNTs) and unmodified graphite. Two different immobilisation methods have been exploited, cross-linking the enzyme on top of the electrode with glutaraldehyde or poly(ethyleneglycol)deglycidyl ether (PEGDGE), which showed different sensitivities and stabilities. After optimisation of the working conditions of the biosensors, they were able to detect lactose in a concentration range between 0.5–200 μM and 0.5–100 μM, with *Tv*CDH and *Ps*CDH, respectively, with a LOD of 250 nM for both. The developed biosensors were successfully tested for the determination of lactose in dairy (milk with different percentages of fat, lactose-free milk and yogurt) with a good reproducibility (RSD = 1.5–2.2%). Improved performance of such lactose biosensors was developed by Tasca and co-workers [111,122]. Most of the work was focused on the electrode modification in order to achieve efficient DET through the electrostatic interactions between the negatively recharged *Ps*CDH and the positively charged electrode surface due to the presence of –NH_2_ groups. In particular, single walled carbon nanotubes (SWNCTs) were surface modified with aryl diazonium salts of *p*-phenylenediamine (NH_2_-PD) and deposited on top of a glassy carbon (GC) electrode [123]. The *Ps*CDH/NH_2_-PD/SWCNTs-GC based lactose biosensor showed very efficient DET and exhibited an extraordinary high current density of 500 μA cm^−2^ in a 5 mM lactose solution at pH 3.5. The biosensor exhibited a detection limit for lactose of 0.5 μM, a large linear range from 1 to 150 μM lactose and a high sensitivity (476.8 nA μM^−1^ cm^−2^). It showed also a fast response time (4 s), good reproducibility (RSD = 1.75%) and good stability (half-life 12 days).

In another approach, Tavahodi and co-workers developed a lactose biosensor based on *Ps*CDH immobilised onto positively charged polyethyleneimine (PEI) modified gold nanoparticles (AuNPs) [101]. In this work, they synthesised PEI@AuNPs by using PEI as reducing agent for Au(III) and as stabiliser for the NPs. Next, the PEI@AuNPs were drop-cast on top of solid planar Au electrodes creating a favourable environment for the correct orientation of *Ps*CDH, due to the strong electrostatic interactions. The heterogeneous electron-transfer (ET) rate (*k*_s_) for the redox reaction of immobilised *Ps*CDH at the modified electrodes was calculated based on the Laviron theory [124] and was found to be 39.6 ± 2.5 s^−1^. The proposed lactose biosensor exhibits a good long term stability as well as a high and reproducible sensitivity to lactose, viz. 3.93 µA mM^−1^, with a response time less than 5 s and a linear range from 1 to 100 µM.

Other nanomaterials for example, palladium (PdNPs) or platinum nanoparticles (PtNPs) in combination with MWCNTs or nanohybrids have been used to enhance the lactose biosensor sensitivity and stability in recent years. In particular, Bozorgzadeh et al. developed a biosensor based on DET of *Pc*CDH immobilised onto PtNPs or PdNPs decorated MWCNTs [112]. The prepared nanohybrids, PtNPs–MWCNTs and PdNPs–MWCNTs, were cast on the surface of spectrographic graphite electrodes and then *Pc*CDH was adsorbed on the modified layer. DET between *Pc*CDH and the modified nanostructured electrodes was studied using flow injection amperometry and cyclic voltammetry. The maximum current responses (*I*_max_) and the apparent Michaelis–Menten constants (K_M_^app^) for the different *Pc*CDH modified electrodes were calculated by fitting the data to the Michaelis–Menten equation and compared. The sensitivity towards lactose was 3.07 and 3.28 µA mM^−1^ at the *Pc*CDH/PtNPs–MWCNTs/SPGE and *Pc*CDH/PdNPs–MWCNTs/SPGE electrodes, respectively, which were higher than those measured at the *Pc*CDH/MWCNTs/SPGE (2.60 µA mM^−1^) and *Pc*CDH/SPGE (0.92 µA mM^−1^).

More recently, Bollella et al. evaluated the influence of an ordered electrode nanostructuration on the sensitivity of a lactose biosensor based on the immobilisation of a class II CDH, *Corynascus thermophilus* CDH (*Ct*CDH) [113]. An efficient DET between *Ct*CDH and the novel gold electrode platform was achieved by covalently linking “green” AuNPs and AgNPs (obtained through a green synthetic pathway using quercetin as reducing agent at room temperature [125]) modified with a dithiol based self-assembled monolayer, consisting of biphenyl-4,4′-dithiol (BPDT). The apparent *k*_S_ of *Ct*CDH was calculated to be 21.5 ± 0.8 s^−1^ and 10.3 ± 0.7 s^−1^, for the AuNPs/BPDT/AuE and the AgNPs/BPDT/AuE platforms, respectively. The modified electrodes were successively used to develop a biosensor for lactose detection. However, the *Ct*CDH/AuNPs/BPDT/AuE based biosensor showed the best analytical performances with an excellent stability, a detection limit of 3 μM, a linear range between 5 and 400 μM and a sensitivity of 27.5 ± 2.5 μA cm^−2^ mM^−1^.

#### 3.1.2. CDH Based Glucose Biosensors

The discovery of class II CDHs also revealed that several of these were also good candidates for making 3rd generation glucose biosensors [103], in contrast to other glucose oxidising dehydrogenases, such as various FAD-glucose dehydrogenases (FAD-GDH) [20], PQQ-glucose dehydrogenase (PQQ-GDH) [27] and GOx that do not show any DET properties [126,127]. Several Class II CDHs were investigated for their catalytic properties and pH profiles [103] and among these especially the following two CDHs, *Myriococcum thermophilum* CDH (*Mt*CDH) [128,129] and *Ct*CDH [53,114,115] have been intensively studied as possible candidates for glucose biosensor making as well as for biofuel cells anodes.

In 2009 the very first paper appeared on the possibility to make 3rd generation glucose sensors based on Class II CDH [130]. In 2011 a glucose biosensors based on DET of *Ct*CDH was published by Tasca et al. [114]. *Ct*CDH was immobilised onto oxidatively shortened SWCNTs through cross-linking the enzyme with PEGDGE. By drop-casting SWCNTs onto the electrode, it was possible to observe a higher sensitivity (222 nA mM^−1^ cm^−2^) compared to a bare graphite electrode and with a linear range between 0.1 and 30 mM and a LOD of 0.05 mM, which covers the whole concentration range relevant for the direct determination of the blood glucose level. With respect to its linear range and detection limit the 3rd generation glucose biosensor might be suited for monitoring the glucose concentration in human blood.

Furthermore, Zafar and co-workers reported on the immobilisation of *Ct*CDH on top of carbon screen-printed electrodes (SPCEs), in order to develop portable DET based glucose biosensor [115]. The enzyme was immobilised on two different commercially available SPCEs, carboxyl-functionalised single-walled carbon nanotubes (SPCE–SWCNTs) and multiwalled carbon nanotubes (SPCE–MWCNTs), by simple physical adsorption or a combination of adsorption followed by cross-linking using with PEGDGE Mn = 400 or glutaraldehyde (GA). The SPCE–MWCNTs/*Ct*CDH/PEGDGE electrode showed the highest sensitivity, a wide linear range (0.025–30 mM) with an LOD of 0.01 mM. Moreover, this biosensor exhibited a high stability by losing only 10% of its initial signal after continuously injecting 50 mM glucose for 7 h.

### 3.2. CDH Based Biofuel Cells

It is known that the development of enzymatic fuel cells (EFCs) recently attracts a growing interest because of the possibility to be used as implantable electric power sources for self-contained biodevices as they can be significantly miniaturised and are also able to operate under human physiological conditions [60,131,132]. Most of the efforts have been addressed to find out the “ideal” sugar oxidising enzyme, able to oxidise both α- and β-D-glucose preferably more than once, insensitive to oxygen and possibly also able to show efficient DET with the enzyme modified electrode [52]. Class II CDHs fulfil some of the aforementioned parameters concerning the “ideal” sugar oxidising enzyme. In this section, the main findings related to EFCs based on DET of class II CDHs as glucose/O_2_ EFCs are summarised (Table 3).

In 2008 Coman et al. published the very first report on a glucose/O_2_ BFC based on DET for both the anode and cathode. In this BFC *Dichomera saubinetii* CDH and *Trametes hirsuta* laccase (*Th*Lac) were both immobilised by simply drop-casting on top of a graphite electrode [133]. The optimum pH of the DET reaction was found close to pH 5, similar to the previously reported DET-optimum pH for class II *Mt*CDH, being in this case coupled with *Th*L, which shows also an acidic optimal pH. The biocatalytic limiting current values of the bioanode vary considerably depending on the sugar, in the order: lactose > cellobiose > glucose. Nevertheless, the EFC exhibited good operational and stability parameters with an open-circuit voltage of 0.73 V, a maximum power density of 5 µWcm^−2^ at 0.5 V and an estimated half-life of more than 38 h in an air-saturated 0.1 M citrate–phosphate buffer at pH 4.5 containing 5 mM glucose. The work was very useful as a proof of concept of the possibility to couple such enzymes through DET based reactions, however, they do not work at human physiological conditions.

Later the same research groups reported on an EFC based on the combination of *Ct*CDH and *Myrothecium verrucaria* bilirubin oxidase (*Mv*BOx) both working nicely at human physiological pH, again simply drop-casting the enzymes on top of graphite electrodes [134]. The mediator-less and membrane-less EFC was tested in phosphate buffer and human serum and showed an open-circuit voltage of 0.62 and 0.58 V and a maximum power density of ca. 3 and 4 µW cm^−2^ at 0.37 and 0.19 V of cell voltage, respectively.

Wang et al. reported on the fabrication and characterisation of a gold-nanoparticle (AuNPs)-based mediatorless sugar/O_2_ EFC operating in neutral sugar-containing buffers and human physiological fluids, such as blood and plasma [53]. In this paper *Ct*CDH and *Mv*BOx were used for making the anode and the cathode, respectively. After a detailed characterisation of each bioelement separately, the *Ct*CDH/AuNPs-based bioanode and the *Mv*BOx/AuNPs-based biocathode were combined into a functional EFC. The following characteristics of the mediator- and membrane-less miniaturised EFC were obtained: an open-circuit voltage of 0.68 V, a maximum power density of 15 µW cm^−2^ at a cell voltage of 0.52 V in phosphate buffer and an open-circuit voltage of 0.65 V and a maximum power density of 3 µW cm^−2^ at a cell voltage of 0.45 V, in human blood. The estimated half-lives of the biodevices were found to be >12, <8 and <2 h in sugar-containing buffer, human plasma and blood, respectively.

More recently, the dimensions of the above reported electrodes were scaled down in order to obtain an EFC operating in basal human lachrymal liquid, which produces enough electrical energy for powering modern low-power electronic devices [50]. Indeed, the following characteristics of miniature glucose/O_2_ biodevices operating in human tears were registered: 0.57 V open-circuit voltage, about 1 µW cm^−2^ maximum power density at a cell voltage of 0.5 V and more than 20 h operational half-life. Theoretical calculations regarding the maximum recoverable electrical energy can be extracted from the biofuel and the biooxidant, glucose and molecular oxygen, each readily available in human lachrymal liquid.

Similarly, the miniaturised EFC has been used also in sweat and saliva to be able to power small electronic devices for biomedical in-situ monitoring, which require a low power output [135]. In this work thin gold wires were used as electrodes, modified according to the protocol reported by Wang et al. [53]. The following characteristics of miniature glucose/O_2_ biodevices were registered in human sweat and saliva, respectively: 580 and 560 mV open-circuit voltage, 0.26 and 0.1 µW cm^−2^ power density at a cell voltage of 0.5 V, with up to ten times higher power output at 0.2 V. When saliva was collected after meal ingestion, roughly a two-fold increase in power output was obtained, with a further two-fold increase by addition of 500 µM glucose. Likewise, the power generated in sweat at 0.5 V increased two-fold by addition of 500 µM glucose.

In a similar approach, Krikstolaityte et al. developed a glucose/O_2_ EFC based on the DET of another class II CDH, *Humicola insolens* CDH (*Hi*CDH), immobilised on positively charged thiol capped AuNPs with N-(6-mercapto)hexylpyridinium (MHP), while the biocathode was still based on *Mv*BOx/AuNPs modified electrode [51]. In this paper, the results achieved in terms of power output were slightly lower compared to the results of the paper reported previously: (i) 5 mM glucose-open-circuit voltage (OCV) of 0.65 V and the maximal power density of 4.77 µW cm^−2^ at an operating voltage of 0.50 V; (ii) 10 mM lactose-OCV of 0.67 V and the maximal power density of 8.64 µW cm^−2^ at an operating voltage of 0.50 V. Conversely, the stability parameters resulted to be higher, indeed the half-life operation times of the EFC were estimated to be at least 13 and 44 h in air saturated PBS containing 5 mM glucose and 10 mM lactose, respectively.

## 4. Fructose Dehydrogenase

Fructose dehydrogenase (FDH; EC 1.1.99.11) is a membrane-bound oxidoreductase found among bacteria belonging to the acetic acid bacteria (AAB) family, a group of gram-negative bacteria able to oxidise various sugars and ethanol with the production of acetic acid during the fermentation process [65]. Among the AAB family, only two genera exhibit oxidizing activity towards D-fructose producing 5-keto-D-fructose, *Acetobacter* and *Gluconobacter* [136]. In particular, *Acetobacter* can oxidise also ethanol to carbon dioxide and water through Krebs cycle enzymes, whereas *Gluconobacter* does not completely oxidise ethanol due to lack of some Krebs cycle enzymes.

FDH belongs to the flavohemoproteins family. It is a heterotrimeric membrane-bound enzyme complex with a molecular mass of 146.4 kDa, consisting of three subunits: subunit I (DH_FDH_), which is the catalytic domain, with a covalently bound FAD cofactor, where D-(-)-fructose is involved in a 2H^+^/2e^−^ oxidation to 5-keto-D-fructose; subunit II (CYT_FDH_), which acts as a built-in electron acceptor with three heme *c* moieties covalently bound to the enzyme scaffold and two of them are involved in the stepwise electron transfer pathway; subunit III, which is not involved in the electron transfer but plays a key role for the enzyme complex stability [48,79,84,137,138].

Unfortunately, the crystal structure of FDH is not available yet, because the enzyme is a membrane bound protein with a high molecular weight (ca. 146 kDa). In the last few years a lot of efforts have been addressed considering new crystallisation methods to solve the problem with membrane bound enzymes. The crystal structure would be a fundamental finding to clarify the electron transfer mechanism of this enzyme with particular attention on the co-factors involved.

**Figure 4:**
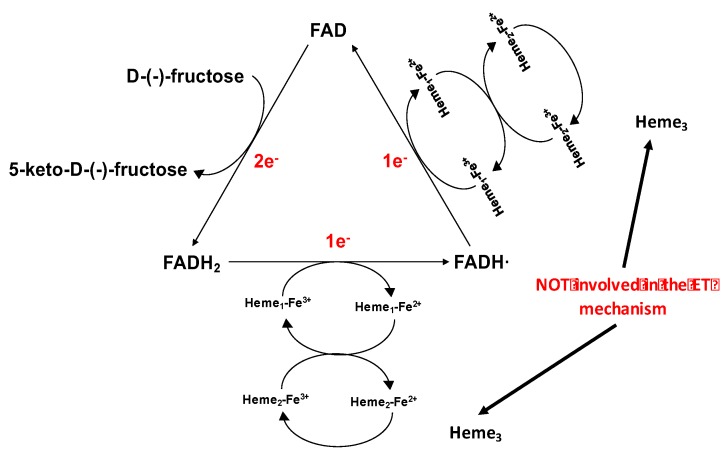
Suggested electron transfer mechanism for fructose dehydrogenase (FDH) at the electrode surface. D-(-)-fructose is oxidised to 5-keto-D-(-)-fructose releasing 2 electrons, which are transferred one by one through FAD first, followed by two heme *c* working as monoelectronic acceptors. Figure 4 is reproduced from [139] published as open-access paper in Analytical and Bioanalytical Chemistry edited by Springer-Verlag.

The ET pathway for FDH immobilised on the electrode surface and in the absence of any competing e^−^ acceptor, is similar as was outlined for CDH above and is assumed to occur as follows (Figure 4) [139]:

(1) D-(-)-fructose is oxidised to 5-keto-D-(-)-fructose involving 2e^−^/2H^+^ with the concomitant reduction of FAD to FADH_2_;
(2) FADH_2_ is sequentially reoxidized in two separate 1 ET steps. In the first FADH_2_ is partially reoxidized to FADH**·** through the IET pathway between the DH_FDH_ and CYT_FDH_ domains, whereby one of the three heme *c* moieties (heme *c*_1_) is reduced. Next, the electron is transferred from heme *c*_1_ to a second heme *c* (heme *c*_2_) of the two hemes involved in the ET pathway and then to a final electron acceptor, which is the electrode when FDH is adsorbed onto the electrode surface;
(3) FADH**·** is finally reoxidized to FAD by heme *c*_1_ and the electron is then transferred to heme *c*_2_ (which gives the second internal electron transfer (IET) step), which in turn is reoxidized by the electrode whereby FDH is returned to its fully oxidised state.

Conversely, in the presence of an electron acceptor such as quinone (Q), the reoxidation of FADH_2_-DH_FDH_ can be accomplished by a 2e^−^/2H^+^ reduction of Q to QH_2_, with a consequently reoxidation of QH_2_ at the electrode surface. Alternatively, in the case of a monoelectronic and non-H^+^ acceptor—for example, an Os^3+^-complex—the electrons are sequentially transferred from the FADH_2_-DH_FDH_ to Os^3+^-complex. Moreover, a second ET from the FADH_2_-DH_FDH_ to a second Os^3+^ complex will fully re-oxidise the DH_FDH_ with the formation of two Os^2+^ complexes at the electrode surface [85,140,141].

Over the past twenty years FDH has been used to develop DET-based fructose biosensors and enzymatic fuel cells less extensively than CDH, probably due to the more restricted application in food technology compared to that in medicine. Glucose, unlike fructose, is an essential energy source in many living organisms and glucose biofuel cells have found applications in powering implantable bioelectronic devices used for diagnose and biomedical applications [70,142]. Nevertheless, the most significant electrochemical platforms will carefully reviewed in following sections.

### 4.1. FDH Based Biosensors

In the earliest article on a fructose biosensor based on DET of FDH, Ikeda et al. reported on the immobilisation of FDH onto a carbon paste electrode [79]. The modified electrode showed an electrocatalytical wave starting at E_ONSET_ = 0 V versus Ag|AgCl_sat_ and rising up to approximately 2.5 µA at 0.7 V versus Ag|AgCl_sat_. The oxidation of D-fructose occurred at the catalytic dehydrogenase domain, from which the electrons were transferred to the second subunit, the cytochrome domain containing the *heme c*:s and finally shuttling the electrons to the electrode [143,144,145,146]. The K_M_ and *I*_max_ values of this electrode were determined as 8.0 ± 1 mM and 1.4 ± 0.4 µA, respectively. Moreover, the FDH biosensor exhibited a linear range between 0.2 and 30 mM.

Afterwards, other papers have been published, where the authors mistakenly believed that the cofactor of the catalytic dehydrogenase domain was PQQ instead of FAD. For example, Parellada et al. reported on the immobilisation of FDH onto polyethyleneimine (PEI) modified carbon paste electrodes [147]. The enzyme was introduced into the carbon paste matrix and the addition of PEI allows an mediatorless anodic current to be achieved at 400 mV versus Ag|AgCl_sat_ after injections of fructose in the FIA system. Operating at 400 mV, the analytical characteristics of the sensor were: limit of detection 75 µM (S/N 3), linearity up to l0 mM, sensitivity 385 µA mM^−1^ cm^−2^, complete specificity for fructose and operational stability of 10 h. Yabuki and co-workers immobilised FDH onto AuNPs modified GC electrodes showing a catalytic current proportional to a concentration of D-fructose up to 0.5 mM [148]. However, the long-term stability of the biosensor was not very good, as the response decreased to 50%, 20% and 10% of the initial current after 1 h, 1 day and 10 days, respectively.

Besides the papers mentioned above, FDH has also been employed by co-immobilising the enzyme with several mediators, like coenzyme Q-6, tetrathiafulvalene (TTF) and tetracyanoquinodimethane (TCNQ), hexacyanoferrate (III) or ferrocene and dihydroxyphenols [145,149,150,151].

Only more recently, several research groups claimed the presence of FAD as co-factor instead of PQQ in subunit I of the enzyme complex, as supposed by the previous works. After that, all researchers referred to FDH as a heterotrimeric flavoprotein-cytochrome *c* complex.

Tominaga and co-workers reported on the development of a novel platform based on FDH directly adsorbed onto CNTs synthesised on a platinum plate by the chemical vapour deposition (CVD) method using iron nanoparticles derived from ferritin [152]. The so modified electrode showed a well-defined electrocatalytical wave from −0.15 V (vs. Ag|AgCl_sat_), which was close to the E°’ of one of the heme *c*’s functioning as prosthetic group of FDH. Moreover, the calibration curve exhibited a linear relationship between current density and fructose concentration up to ca. 40 mM. The Michaelis-Menten constant (K_M_) was found to be 11 ± 1 mM.

In 2014, Nazaruk et al. used liquid crystalline cubic phase (LCP) and CNTs for the immobilisation of FDH in order to enhance the DET of FDH, exploiting the possibility to retain native conformation and bioactivity of FDH [153]. The calibration curve of FDH/LPC/SWCNTs/GC electrode showed a linear relationship between current density and fructose concentration up to 10 mM. The Michaelis-Menten constant (K_M_) was found to be 11 ± 1 mM. The biosensor was tested for fructose detection in fruit juices by using both cyclic voltammetry and amperometry at a constant applied potential. Moreover, the same modified electrode has been used also as bioanode in combination with *Trametes hirsuta* laccase (*Th*Lac) to realise an EFC, of which the performance is reviewed in the next section.

More recently, Šakinytė et al. reported on the immobilisation of FDH onto thermally reduced graphene oxide (TRGO) obtaining three different fractions, namely TRGO1, TRGO2 and TRGO3 [154]. All three TRGO fractions were applicable for design of amperometric fructose biosensors acting on DET principles. The achieved high values of the sensitivity are in the same order of magnitude as for other fructose biosensors based on synergistic mediated processes. The electrode based on TRGO1 exhibited the highest sensitivity of 14.5 μA mM^−1^ cm^−2^, with a LOD of 0.7 mM. In this regard, the functional groups present in the TRGO1 fraction are able not only to orient FDH properly but they can also participate in the electron transfer reactions. In this case, the surface area of the electrode material in bioelectrocatalysis based on DET does not play any crucial role in the effectivity of the ET.

Siepenkoetter and co-workers studied the effect of nanoporous gold with different pores sizes on the bioelectrocatalytical behaviour of FDH in order to develop a highly sensitive and selective biosensor for the detection of fructose. In fact, they synthesised nanoporous gold with pore diameters of 9, 18, 42 and 62 nm by using a de-alloying method [155]. From the preliminary results, the 42 nm-nanoporous gold electrode showed the best performance in terms of catalytic current. Moreover, the so prepared electrode was modified following two different methods: self-assembling monolayer (SAM) of 3-mercaptopropionic acid (3-MPA) and a mixed layer based on 3-MPA SAM with the electrodeposition of 2-carboxy-6-naphthoyl diazonium salt. Finally, FDH was cross-linked onto the modified electrode using carbodiimide (CMC) in a manner that enables DET to occur. The latter modified electrode exhibited the highest electrocatalytical current. So far the response of the biosensor correlated very well with the results obtained with a spectrophotometric commercially available enzymatic kit. The biosensor demonstrated rapid response times (less than 5 s), with a linear range of 0.05–0.3 mM D-fructose, a sensitivity of 3.7 µA cm^−2^ µM and a limit of detection of 1.2 µM, with high selectivity. The biosensor is a promising alternative to established analytical measurements for the detection of fructose.

The main analytical parameters of the reported fructose biosensors based on DET of FDH are summarised in Table 4.

### 4.2. FDH Based Biofuel Cells

In early research into FDH based BFCs, Kamitaka et al. [47] realised a one-compartment biofuel cell, in which FDH from *Gluconobacter* sp. and laccase from *Trametes* sp. (*Ts*Lac) were used as DET-type bioelectrocatalysts in the two-electron oxidation of D-fructose and the four-electron reduction of O_2_ as fuels, respectively. In particular, FDH was drop-cast on top of carbon paper (CP) electrodes modified with Ketjen black (KB) particles, which resulted in a tight immobilisation because of the strong π-π stacking interactions. On the other hand, *Ts*Lac was immobilised onto carbon aerogel (CG) particles with an average pore size of 22 nm, modified on CP electrodes. Both elements resulted in an efficient bioelectrocatalysis, with a maximum current density of 4 mA cm^−2^. The FDH adsorbed KB-modified CP electrodes and the *Ts*Lac-adsorbed CG-modified CP electrodes were combined to construct a one-compartment BFC without separators with an open-circuit voltage of 790 mV, a maximum current density of 2.8 mA cm^−2^ and a maximum power density of 850 mW cm^−2^ at 410 mV of the cell voltage under stirring.

A similar fructose/O_2_ biofuel cell based on DET was realised by Murata et al. [156] because of the large surface area of the electrodes it was possible to increase the current density during the bioelectrocatalysis. AuNPs were synthesised according to a citrate protocol and successively directly drop-cast to immobilise *Mv*BOx, for the biocathode and modified through a SAM reaction with mercaptoethanol (ME) to have a positive influence on the immobilisation of FDH, for the bioanode. The BOx-adsorbed AuNPs/CP and FDH-adsorbed ME-AuNPs/CP electrodes were combined to construct a mediatorless BFC without separator. The following characteristics of the mediator-, separator- and membrane-less BFC were obtained: a maximum current density of 2.6 mA cm^−2^, a maximum power density of 0.66 mW cm^−2^ at 360 mV of the cell voltage in quiescent solution while under stirring, a maximum current density of 4.9 mA cm^−2^ and a maximum power density of 0.87 mW cm^−2^ at an operating voltage of 300 mV.

More recently, Haneda et al. prepared a totally flexible, sheet shaped BFC by using a carbon fabric (CF) as the flexible, conductive base for the enzyme electrodes. The CF strips were modified with MWCNTs and FDH for the oxidation of fructose and with Ketjien black (KB) and *Mv*BOx for the reduction of oxygen. The FDH anode strip and the BOx cathode strip were stacked with a hydrogel film of agarose, which retains the electrolyte solution and fuel (fructose). This assembly provides a stand-alone, sheet-shaped power source that can be bent without loss of output power. The open-circuit voltage of the cell was found to be 0.7 V with a maximum power density reaching 550 µW cm^−2^ at 0.4 V. It is interesting to note that this device could be repeatedly bent up to a 44° angle without significant loss of output power, while bending in excess of this value would cause fracture of the modified electrodes [157].

In a similar approach, Miyake et al. [158] described a layered BFC constructed by laminating enzyme-modified carbon fabric (CF) strips and hydrogel film containing electrolyte and fuel. The hydrogel sheets ensure ion-conduction between anode/cathode fabrics and also serve as the fuel tank to eliminate the necessity of packaging. This device resulted in having a better performance compared to that previously developed by Haneda et al. because of the different and improved hydrogel. Using agarose resulted in a thick layer but really weak and not resistive to continuous bending stress. Conversely, Miyake and co-workers used a heavy-duty “double network (DN) hydrogel,” resulting in a very flexible, thinner BFC (~1 mm thickness). The pre-modification of CF with CNTs was effective to improve the performance of both bioelements, with a maximum power of 0.64 mW at 1.21 V and an open circuit voltage of 2.09 V.

In 2014, Nazaruk et al. [153] used monoolein or phytantriol liquid crystalline cubic phase and CNTs for the immobilisation of FDH. Entrapment within the cubic phase prevents the guest protein from chemical and physical degradation, thus facilitating retention of its native conformation and bioactivity. The mesophase environment was therefore found appropriate for retaining FDH close to the electrode surface. Phytantriol was used as the cubic phase component in case of measurements carried out in biological fluids containing hydrolysing enzymes. The enzymatic fuel cell based on FDH in the cubic phase film at the anode and *Cerrena unicolor* C-139 laccase at the biocathode showed open circuit potential of 703 ± 10 mV in the presence of 40 mM fructose and power density of 850 µW cm^−2^ at 250 mV under continuous flow of O_2_.

In the same year, So and co-workers tried to improve the performances of a DET-type fructose/O_2_ BFC with a substrate modified biocathode. The hypothesis was that when the electrode is modified with adsorbed bilirubin, a substrate for *Mv*BOx, the enzyme would attractively interact with the modified compound in such an orientation that the electron-accepting T1 site faces the electrode surface. The orientation seems to be convenient for the DET-type bioelectrocatalysis. The authors also attempted to create a high performance bioanode optimising the conditions for adsorbing FDH on carbon cryogel (CCG) electrodes, a mesoporous carbon material. Finally, a one-compartment DET-type BFC without separators was realised, an open-circuit voltage of 0.79 V and a maximum power density of 2.6 mW cm^−2^ at 0.46 V were achieved without stirring and under air atmospheric conditions [49].

In the most recent paper for a fructose/O_2_ BFC, Kizling and co-workers [159] employed an eco-friendly cellulose/polypyrrole composite to immobilise FDH in order to obtain a bioanode working at a more negative potential thus affecting the operational parameters of the BFC. The modified bioanode was combined with a *Th*Lac based biocathode. In this case, laccase was immobilised onto naphthoquinone-modified CNTs. This work was aiming at increasing the capacitance of both electrodes in order to possibly use the final device as a biosupercapacitor. The BFC with the GCE cathode covered with laccase adsorbed on naphthylated MWCNTs exhibits improved parameters with an open circuit voltage of 0.76 V and a maximum power density of 1.6 mWcm^−2^ at cell voltage of 0.33 V.

Table 5 summarises the most important characteristics of the mentioned fructose/O_2_ BFCs based on DET of FDH in the bioanode.

## 5. Enzyme Engineering to Enhance DET for Future Biosensors and BFC Developments

One of the most promising approaches to improve bioelectrocatalytic reactions based on DET of dehydrogenases is the production of mutated or modified enzymes through the use of genetic engineering and recombinant technology, both of which have opened the doors to widespread exploitations of new dehydrogenases for sensing devices with desired electrochemical properties [160,161,162]. In particular, engineered enzymes enabled to realise DET-based biodevices with enhanced sensitivity and wider linear range and with extended stability, as shown in the papers referred to below.

Sode and co-workers performed a series of mutations on PQQ-glucose dehydrogenase extracted from different bacteria and realised glucose biosensors with improved performances. In one of the earlier reports, they used engineered *Escherichia coli* PQQ-glucose dehydrogenase, where a histidine residue, His775, was exchanged for aspartate (Asp). This mutant exhibited a 25-fold higher K_M_, thus showing an extended linear range for the glucose biosensor (3–70 mM) compared to the colorimetric test (0.5–30 mM) [163]. In another work they fused the cytochrome domain of PQQ-alcohol dehydrogenase to the C-terminal of PQQ-glucose dehydrogenase from *Acinetobacter calcoaceticus* and the constructed fused protein showed very good electron transfer properties from the heme to the electrode, allowing the construction of a glucose biosensor with higher sensitivity [164].

Yuhashi et al. [163] demonstrated that the use of suitable mutants can improve the stability of BFCs. They constructed a BFC employing an engineered PQQ-glucose dehydrogenase, the SER415Cys mutant, from *Acinetobacter calcoaceticus* as the bioanode enzyme and BOx as the biocathode enzyme. The lifetime of the system was greatly enhanced up to 152 h, more than six times than that of the BFC employing the wild-type enzyme. Recently a FAD-dependent glucose dehydrogenase was fused to a natural minimal cytochrome domain in its C-terminus to achieve DET [165] revealing that through protein engineering DET properties of the enzyme can be obtained.

As for FAD-dependent CDH and FDH, we have reported several investigations, where the enzymes have been shown to undergo internal ET to promote direct bioelectrocatalysis but also in these cases enzyme engineering has been utilised to improve the selectivity, performance and stability of the bioelectrodes for both biosensors and BFCs, as illustrated in the following examples.

Both *Ct*CDH and *Hi*CDH have been altered by a single cysteine to tyrosine substitution in position 291 and 285 of the active sites of *Ct*CDH and *Hi*CDH, respectively. The engineering modification resulted in improved kinetic constants for glucose and a concomitant decrease in activity for several disaccharides, including maltose, with a consequent higher glucose sensitivity and reduced maltose affinity of the biosensor [166,167,168].

Hibino et al. [169] constructed a variant of FDH that lacks 143 amino acid residues. The downsized engineered protein caused an increase in the surface concentration of the electrochemically effective enzyme molecules and an improvement in the heterogeneous electron transfer kinetics. This study is the first trial of the deletion of such a long region that includes one of the prosthetic groups to enhance the direct electrocatalysis of DET-based devices such as biosensor and BFCs. Drastic increases in current densities and sensitivity for lactose could also be obtained by deglycosylating *Phanerochaete chrysosporium* (*Pc*CDH) and *Ceriporiopsis subvermispora* (*Cs*CDH) as a result of both increasing *k*_s_ as well as the number of enzyme molecules on the electrode surface [170].

Although the electrochemical properties of many dehydrogenases have been improved by protein engineering, the coupling of such engineered enzyme with various nanomaterial based modified electrodes still represents an important tool to facilitate the electron transfer between enzyme and electrode.

Nanomaterial-facilitated protein DET has been extensively studied in recent years in order to explore the fundamental insights in the effects of nanomaterial properties on protein DET behaviours. Research aims to design and fabricate novel nanocomposites with desirable properties to enhance protein DET capacity for the development of high-performance bioelectrochemical devices. However, the further enhancement of the DET capacity necessary to develop high-performance biosensors and BFCs for practical applications remains a great challenge.

## 6. Conclusions

Direct electron transfer between dehydrogenase and electrode is a very promising approach to obtain high performance biosensors and BFCs. Unfortunately, only a restricted number of dehydrogenases show the phenomenon of DET on electrode surfaces.

Although many efforts have been carried out to elucidate the direct electrocatalysis of dehydrogenases, the mechanism of the DET is not thoroughly understood yet. There are some questions that need to be clarified: (i) what is the exact role of the enzyme prosthetic group in the electrocatalysis and in the electron transfer process; (ii) what is the influence of the nature of the electrode material and the structure of the electrode surface; (iii) the relationship between the distance separating the active site of the enzyme and the electrode surface and the efficiency of the electrocatalysis.

Despite these difficulties, researchers are exploiting two strategies in order to improve the DET efficiency of dehydrogenases: the design and development of new nanomaterial-based electrode platforms and of novel surface immobilisation methods and the synthesis of new engineered redox proteins/enzymes, achieved through gene and protein engineering techniques, resulting in a manipulation of their redox potentials and an increase in electron-transfer efficiency.

The use of both nanomaterials and engineered dehydrogenases with desired properties may led to a broad spectrum of practical applications ranging from biosensors to BFCs with improved performances.

Another important advantage of the direct electrocatalysis of dehydrogenases is the possibility of miniaturisation of the sensing elements and the development of implantable devices. However, further research into improving the stability and performance are still needed to improve sensitivity, operational lifetimes and open circuit potentials of the DET-dehydrogenase-based electrochemical devices.

## Figures and Tables

**Table 1:** Multicofactor oxidases/dehydrogenases for which Direct Electron Transfer (DET) reactions with electrodes have been shown.

Dehydrogenases	Cofactor	Substrate	Ref.
Cellobiose dehydrogenase	FAD-heme	D-glucose, cellobiose, lactose	[44,45,82,83]
D-Fructose dehydrogenase	FAD-heme	D-fructose	[46,49,84,85]
Pyranose dehydrogenase	FAD-heme	aldoses	[33]
Lactate dehydrogenase	PQQ-heme	lactate	[86]
Lactate dehydrogenase/cyt *b*_2_	FMN-heme	lactate	[41]
Alcohol dehydrogenase	PQQ-heme	ethanol	[30,87]
Succinate dehydrogenase	FAD-Fe-S cluster-heme	succinate	[80,81]
D-gluconate dehydrogenase	FAD-heme-Fe-S cluster	D-gluconate	[43,88]
D-glucose dehydrogenase	FAD-heme-Fe-S cluster	D-glucose	[89]
Aldose dehydrogenase	PQQ-heme	D-glucose	[90]
Pyruvate dehydrogenase	PQQ-heme	pyruvate	[91]
Aldehyde dehydrogenase	PQQ-heme	aldehyde	[92]
Sulphite oxidase	Moco-heme	sulphite	[35,39]
Sulphite dehydrogenase	Moco-heme	sulphite	[42]
Theophylline oxidase	?-heme	theophylline	[34,36,38]

**Table 2:** Lactose (upper part) and glucose biosensors (bottom part) based on DET of CDH are compared based on several analytical parameters such as linear range, LOD, sensitivity, CDH class, applied potential and stability. Abbreviations: AuE gold electrode, AuNPs gold nanoparticles, *Ct*CDH *Corynascus thermophilus* CDH, GC glassy carbon electrode, MWCNTs multi-walled carbon nanotubes, NH_2_-PD aryl diazonium salts of *p*-phenylenediamine, *Pc*CDH *Phanerochaete chrysosporium* CDH, PdNPs palladium nanoparticles, PEDGE poly(ethylene glycol) diglycidyl ether, PEI polyethyleneimine, *Ps*CDH *Phanerochaete sordida* CDH, PtNPs platinum nanoparticles, SPE screen printed electrode, SPGE spectrographic graphite electrode, SWCNTs single-walled carbon nanotubes, *Tv*CDH *Trametes villosa* CDH.

Lactose Biosensors							
Electrode Platforms	Linear Range/(µM)	LOD/(µM)	Sensitivity/(µA mM^−1^ cm^−2^)	Class	Applied Potential/V vs. Ag|AgCl_sat_	Stability	Ref.
*Tv*CDH/PEDGE/MWCNTs/SPE	0.5–200	0.25	-	I	+0.198	100% of initial response after 8 h	[110]
*Ps*CDH/PEDGE/MWCNTs/SPE	0.5–100			I			
*Ps*CDH/NH_2_-PD/SWCNTs-GC	1–150	0.5	476.8	I	+0.2	85% of the initial response after 50 h	[111]
*Ps*CDH/ PEI@AuNPs/AuE	1–100	0.3	196.5	I	+0.25	95% of the initial response after 24 h	[101]
*Pc*CDH/PtNPs–MWCNTs/SPGE	-	-	43.5	I	+0.29	75% of their initial response after 10 h	[112]
*Pc*CDH/PdNPs–MWCNTs/SPGE	-	-	46.4	I	+0.29		
*Ct*CDH/AuNPs/BPDT/AuE	5–400	3	27.5	II	+0.25	85% of initial response after 20 days	[113]
**Glucose Biosensors**							
*Ct*CDH/PEDGE/MWCNTs/GC	0.1–30	0.05	222	II	+0.190	-	[114]
*Ct*CDH/PEDGE/MWCNTs-SPE	0.025–30	0.01	-	II	+0.198	90% of initial response after 7 h	[115]
*Ct*CDH/PEDGE/SWCNTs-SPE	0.025–30	0.01		II			

**Table 3:** DET-based lactose/glucose and glucose/oxygen enzymatic fuel cells (EFCs) are compared based on some of the operational parameters like operating conditions, open-circuit voltage (OCV), power output and operational stability. Abbreviations: AuE gold electrode, AuMWs gold microwires electrode, AuNPs gold nanoparticles, *Ct*CDH *Corynascus thermophilus* CDH, *Dc*CDH *Dichomera saubinetii* CDH, *Hi*CDH *Humicola insolens* CDH, MHP N-(6-mercapto)hexylpyridinium, *Mv*BOx *Myrothecium verrucaria* bilirubin oxidase, SPGE graphite electrode, *Th*Lac *Trametes hirsuta* laccase.

BFC	Conditions	OCV/(V)	Power Output/Limiting Element (l.e.)	Operational Stability	Ref.
*Dc*CDH/*Th*Lac SPGE-based	100 mM citrate–phosphate air-saturated buffer, pH 4.5 containing 5 mM glucose	0.73	5 µW cm^−2^ at 0.5 V (l.e.: anode)	Half-life > 38 h	[133]
*Ct*CDH/*Mv*BOx SPGE-based	50 mM PBS buffer pH 7.4 containing 5 mM glucose and 150 mM NaCl	0.62	~3 µW cm^−2^ at 0.37 V (l.e.: anode)	Half-life > 6 h	[134]
	Human serum	0.58	~4 µW cm^−2^ at 0.19 V (l.e. cathode)	Half-life < 2 h	
*Ct*CDH/*Mv*BOx AuNPs/AuE-based	50 mM PBS buffer air-saturated pH 7.4 containing 5 mM glucose and 150 mM NaCl	0.68	3.3 µW cm^−2^ at 0.52 V (l.e. anode)	~20% drop in 12 h of continuous operation	[53]
	50 mM PBS buffer air saturated pH 7.4 containing 5 mM lactose	0.68	14.9 µW cm^−2^ at 0.52 V (l.e. anode)	Half-life > 12 h	
	Human blood	0.66	2.8 µW cm^−2^ at 0.45 V (l.e. cathode)	Half-life < 3 h	
	Human plasma	0.63	3 µW cm^−2^ at 0.47 V (l.e. cathode)	Half-life < 8 h	
*Ct*CDH/*Mv*BOx AuNPs/AuE-based (contact lenses)	Human tears	0.57	1 µW cm^−2^ at 0.5 V (l.e. cathode)	Half-life > 20 h	[50]
*Ct*CDH/*Mv*BOx AuNPs/AuMWs-based	Sweat	0.58	0.26 µW cm^−2^ at 0.5 V (l.e. cathode)	Half-life > 10 h	[135]
	Sweat + 500 µM glucose	0.61	0.47 µW cm^−2^ at 0.5 V (l.e. cathode)	-	
	Saliva before lunch	0.56	0.1 µW cm^−2^ at 0.5 V (l.e. cathode)	-	
	Saliva after lunch	0.56	0.2 µW cm^−2^ at 0.5 V (l.e. cathode)	-	
	Saliva after lunch + 500 µM glucose	0.60	0.46 µW cm^−2^ at 0.5 V (l.e. cathode)	-	
*Hi*CDH/MHP/AuNPs/AuE-based *Mv*BOx AuNPs/AuE-based	50 mM PBS air-saturated buffer pH 7.4 containing 5 mM glucose	0.65	4.77 µW cm^−2^ at 0.50 V (l.e. anode)	Half-life > 13 h	[51]
	50 mM PBS air-saturated buffer pH 7.4 containing 10 mM lactose	0.67	8.64 µW cm^−2^ at 0.50 V (l.e. anode)	Half-life > 44 h	

**Table 4:** Fructose biosensors based on DET of FDH are compared based on several analytical parameters like linear range, LOD, sensitivity, applied potential and stability. Abbreviations: Aunanoporous gold nanoporous, AuNPs gold nanoparticles, CP carbon paste, FDH fructose dehydrogenase, GC glassy carbon electrode, LCP lipidic cubic phase, MPA 3-mercaptopropionic acid, MWCNTs multiwalled carbon nanotubes, NPD 2-carboxy-6-naphtoyl diazonium salt, PEI polyethyleneimine, SWCNTs single-walled carbon nanotubes, TRGO1 thermally reduced graphene oxide 1.

Fructose Biosensors					
Electrode Platforms	Linear Range/(mM)	LOD/(mM)	Sensitivity/(µA mM^−1^ cm^−2^)	Applied Potential/V vs. Ag|AgCl_sat_	Ref.
FDH/CP	0.2–30	-	-	+0.2	[79]
FDH/PEI/CP	Up to 10	75	385	+0.4	[147]
FDH/AuNPs/GC	Up to 0.5	-	-	+0.5	[148]
FDH/MWCNTs/GC	Up to 40	5	-	-	[152]
FDH/LCP/SWCNTs/GC	Up to 10	-	4	+ 0.2	[153]
FDH/TRGO1/GC	0.7–8.8	0.7	14.5	+ 0.4	[154]
FDH/MPA-NPD/Aunanoporous	0.05–0.3	0.0012	3.7	+ 0.15	[155]

**Table 5:** DET-based fructose/O_2_ EFCs are compared based on some of the operational parameters like operating conditions, open-circuit voltage (OCV), power output and operational stability. Abbreviations: AuNPs gold nanoparticles, CCG carbon cryogel, cell. Cellulose, CG carbon aerogel, CP carbon paper electrodes, FDH fructose dehydrogenase, KB Ketjen black particles, LCP lipidic cubic phase, ME mercaptoethanol, *Mv*BOx *Myrothecium verrucaria* bilirubine oxidase, MWCNTs multi-walled carbon nanotubes, NQ naphthoquinone, PPy polypyrrole, SWCNTs single-walled carbon nanotubes, *Th*Lac *Trametes hirsuta* laccase, *Ts*Lac *Trametes* sp. laccase.

BFC	Conditions	OCV (V)	Power Output/Limiting Element	References
FDH/KB/CP *Ts*Lac/CG/CP	0.1 M McIlvaine O_2_-satured buffer (pH 5.0) containing 200 mM fructose	0.79	850 mW cm^−2^ at 0.41 V under stirring (l.e.: cathode)	[47]
FDH/ME-AuNPs/CP *Mv*BOx/AuNPs/CP	0.1 M acetate O_2_-satured buffer (pH 6.0) containing 200 mM fructose	0.73	0.66 mW cm^−2^ at 0.36 V without stirring (l.e.: cathode)	[156]
			0.87 mW cm^−2^ at 0.3 V under stirring (l.e.: anode)	
FDH/*Mv*BOx KB-sheet shaped electrodes	0.15 M McIlvaine O_2_-satured buffer solution (pH 5.0) containing 200 mM fructose.	0.70	0.55 mW cm^−2^ at 0.4 V (l.e.: cathode)	[157]
FDH/*Mv*BOx KB-carbon strips electrodes	0.25 M McIlvaine O_2_-satured buffer solution (pH 5.0) containing 500 mM fructose	2.09	0.64 mW at 1.2 V (l.e.: cathode)	[158]
FDH/*Th*Lac LCP-SWCNTs based GC	0.15 M McIlvaine O_2_-satured buffer (pH 5.0) containing 40 mM fructose	0.70	0.85 mW cm^−2^ at 0.25 V under stirring (l.e.: anode)	[153]
FDH/*Mv*BOx CCG based electrodes	1 M citrate O_2_-satured buffer (pH 5.0) containing 500 mM fructose	0.79	2.6 mW cm^−2^ at 0.46 V (l.e: cathode)	[49]
FDH/cell./PPy/MWCNTs/GC ThLac/NQ/MWCNTs/GC	0.1 M McIlvaine O_2_-satured buffer solution (pH 5.3) containing 100 mM fructose	0.76	1.6 mW cm^−2^ at 0.33 V (l.e.: cathode)	[159]
